# A Rare Duplication on Chromosome 16p11.2 Is Identified in Patients with Psychosis in Alzheimer's Disease

**DOI:** 10.1371/journal.pone.0111462

**Published:** 2014-11-07

**Authors:** Xiaojing Zheng, F. Yesim Demirci, M. Michael Barmada, Gale A. Richardson, Oscar L. Lopez, Robert A. Sweet, M. Ilyas Kamboh, Eleanor Feingold

**Affiliations:** 1 Department of Biostatistics, Graduate School of Public Health, University of Pittsburgh, Pittsburgh, Pennsylvania, United States of America; 2 Department of Human Genetics, Graduate School of Public Health, University of Pittsburgh, Pittsburgh, Pennsylvania, United States of America; 3 Department of Psychiatry, School of Medicine, University of Pittsburgh, Pittsburgh, Pennsylvania, United States of America; 4 Department of Neurology, School of Medicine, University of Pittsburgh, Pittsburgh, Pennsylvania, United States of America; 5 VISN 4 Mental Illness Research, Education and Clinical Center, VA Pittsburgh Healthcare System, Pittsburgh, Pennsylvania, United States of America; 6 Department of Epidemiology, Graduate School of Public Health, University of Pittsburgh, Pittsburgh, Pennsylvania, United States of America; 7 Department of Pediatrics, School of Medicine, University of North Carolina, Chapel Hill, North Carolina, United States of America; UTHSCSH, United States of America

## Abstract

Epidemiological and genetic studies suggest that schizophrenia and autism may share genetic links. Besides common single nucleotide polymorphisms, recent data suggest that some rare copy number variants (CNVs) are risk factors for both disorders. Because we have previously found that schizophrenia and psychosis in Alzheimer's disease (AD+P) share some genetic risk, we investigated whether CNVs reported in schizophrenia and autism are also linked to AD+P. We searched for CNVs associated with AD+P in 7 recurrent CNV regions that have been previously identified across autism and schizophrenia, using the Illumina HumanOmni1-Quad BeadChip. A chromosome 16p11.2 duplication CNV (chr16: 29,554,843-30,105,652) was identified in 2 of 440 AD+P subjects, but not in 136 AD subjects without psychosis, or in 593 AD subjects with intermediate psychosis status, or in 855 non-AD individuals. The frequency of this duplication CNV in AD+P (0.46%) was similar to that reported previously in schizophrenia (0.46%). This duplication CNV was further validated using the NanoString nCounter CNV Custom CodeSets. The 16p11.2 duplication has been associated with developmental delay, intellectual disability, behavioral problems, autism, schizophrenia (SCZ), and bipolar disorder. These two AD+P patients had no personal of, nor any identified family history of, SCZ, bipolar disorder and autism. To the best of our knowledge, our case report is the first suggestion that 16p11.2 duplication is also linked to AD+P. Although rare, this CNV may have an important role in the development of psychosis.

## Introduction

About 40–60% of patients with late-onset Alzheimer's disease (AD), defined by an onset after age 60 years, develop psychosis (AD+P) [1], [2]. The estimated heritability of AD+P is about 61% [3]. Recent large scale genome-wide association study (GWAS) of Alzheimer's disease with psychotic symptoms [3] found several single nucleotide polymorphisms (SNPs) that were tentatively associated with AD+P when compared to AD-P, but none of them were genome-wide significant. Some of these associations were overlapped with other psychiatric disorders with psychotic features such as schizophrenia (SCZ). Several other studies also reported some susceptibility loci and candidate genes for AD+P which increased the risk of schizophrenia (SCZ) and other psychiatric disorders, although the specific genetic determinants remain to be identified [1], [4]–[6].

One type of genetic variant that has been of great interest in the field of psychiatric disorders in the past few years is DNA copy number [7]–[10]. Since GWAS did not identify SNPs that can explain the high heritability of AD+P, rare structural variation such as CNVs may play a role and account for missing heritability. Recent studies have shown that autism and SCZ share several rare copy number variants (CNVs) [10], [11]. Some of these CNVs are also shared with various intellectual disability syndromes [12]. Moreno-De-Luca et al [13] summarized CNV studies in autism and SCZ, and reported 7 recurrent CNVs across autism and SCZ, which included a deletion on chromosome 1q21.1 (position in human genome build 36/hg18: 144,963,732-145,864,377), a deletion on chromosome 3q29 (197,244,288-198,830,238), a deletion on chromosome 15q13.3 (28,698,632-30,234,007), a duplication on chromosome 16p11.2 (29,557,553-30,107,434), a duplication on chromosome 16p13.11 (15,421,876-16,200,195), a deletion on chromosome 17q12 (31,893,783-33,277,865) and a deletion on chromosome 22q11.2 (17,412,646-19,797,314). These 7 CNVs share two features: large size and rare frequency. Each of these CNV regions contains tens to hundreds of genes and their frequencies in cases and controls were about 0.5%, and 0.05%, respectively. We designed this study to examine whether AD+P shares risk CNVs with autism and SCZ. We specifically searched for CNVs for AD+P in the above 7 CNV regions previously reported to be shared across autism and SCZ, using the Illumina HumanOmni1-Quad BeadChip (San Diego, CA, USA).

## Materials and Methods

### Subjects

AD cases and controls were recruited through the University of Pittsburgh Alzheimer's Disease Research Center. Controls met the criteria for being free of dementia using the Mini Mental State Exam. Cases were those with diagnosis of either probable or definite AD according to criteria set by the National Institute of Neurological and Communicative Disorders and Stroke – Alzheimer's disease and Related Disorders Association [14] and the Consortium to Establish a Registry for Alzheimer's Disease (CERAD). All cases have age of onset of at least 60 years. Subjects with evidence of persistent and or recurrent delusions or hallucinations, defined by the CERAD behavior rating scale [15] items for psychotic features (items 33–35) rated as occurring three or more times in the past month at any visit, were classified as AD+P, as previously described [16] Subjects with evidence of infrequent distortions of thought or perception, defined by any of these items rated as occurring 1 to 2 times in the past month, were classified as "indeterminate psychosis” as such symptoms can result from a variety of sources of phenocopies. Subjects rated as no occurrence of possible psychotic symptoms at all visits were classified as AD-P. Subjects were excluded if they had a previous history of SCZ or bipolar disorder. The University of Pittsburgh ADRC follows a standard evaluation protocol, including medical history, general medical and neurological examinations, psychiatric interview, neuropsychological testing and MRI scan. All subjects were recruited with written informed consent, and the study was approved by the University of Pittsburgh Institutional Review Board.

### Genotyping

All subjects were genotyped using the Illumina HumanOmni1-Quad BeadChip. Table 1 summarizes the number of individuals in different categories. After quality control, 2024 samples were retained for the final analysis. Details on the study population characteristics and the quality control parameters applied on the samples and markers can be found elsewhere [17], [18]. Briefly, samples were filtered if they had a missing genotype rate ≥0.02, or with mean X-chromosome heterozygosity ≥0.02 for males or outside the range of 0.25∼0.4 for females. Genetic outliers and those with evidence of relatedness (IBD estimate ≥0.4) or non-European ancestry based on genotype data were also excluded. After filtering, 496 AD+P cases, 639 AD intermediate P, 156 AD−P cases and 958 unaffected controls were retained. Markers were excluded if they had a genotype missing rate of >0.02. Markers were examined to determine if missing depended on case/control status or the genotyping batch.

**Table 1:** Sample sizes for each study group before and after filtering by LRR (Log R Ratio) deviation.

	AD+P	AD intermediate P	AD-P	Non-AD controls
Before filtering	496	639	156	958
After filtering	440	593	136	855

AD: Alzheimer's disease; AD+P: subjects with psychosis in Alzheimer's disease; AD-P: subjects with Alzheimer's disease without psychosis.

### Detection of CNVs

We generated CNV calls using the PennCNV software (2009 Aug27 version) [19]. PennCNV is a Hidden Markov Model (HMM) based method. It uses the log R ratio (LRR) and B allele frequency (BAF) measures computed from the signal intensity files by Beadstudio to detect the CNVs. We used the GC model wave adjustment procedure in PennCNV. After GC model adjustment, we filtered the samples that met the criterion of LRR standard deviation ≥0.3, and |GC base pair wave factor (GCWF)| >0.05 (Table 1). All procedures followed the user guidelines of PennCNV. PennCNV inferred the copy number at each marker, and called a CNV when copy number changes in 3 or more consecutive markers. CNVs with copy number >2 were defined as duplications; while those with copy number <2 were considered deletions. We specifically searched for rare CNVs in the 7 recurrent CNV regions identified across autism spectrum disorders (ASD) and SCZ and summarized by Moreno-De-Luca et al [13]. We plotted the number of individuals who carried CNVs in those 7 regions by different sub-groups (AD+P, AD indeterminate P, AD- P, and no-AD). We also drew the LRR and BAF plots for each of the identified CNV carriers. The significance of association between CNVs and AD+P was measured by Fisher's exact test. All data analysis was programmed in R (2.15.2) [20], except for power analysis which was completed using G*Power (3.0.10) [21]. Human NCBI Build 36 (hg18) was used for this study.

### Verification of predicted CNVs

We validated our CNV findings using the NanoString nCounter CNV Custom CodeSets (Seattle, WA, USA) and following the manufacturer's protocol (Genomics and Proteomics Core Laboratories, University of Pittsburgh). We tested 46 samples in the validation study: two AD+P subjects with predicted 16p11.2 duplication CNV; six AD+P subjects without CNVs; three AD subjects with indeterminate psychosis; one AD-P subject; and 34 non-AD individuals. In addition, two replicates were included in the analysis in order to test the consistency of our findings. A total of 10 CNV probe pairs were designed targeting the region of interest on chromosome 16 (from 29,554,843 bp to 30,105,652 bp). Briefly, Genomic DNAs were fragmented into small pieces (200–800 bp) and denatured to produce single strands. The fragmented, denatured DNA samples were then hybridized to the Custom CNV CodeSets, each containing a capture probe and a reporter probe tagged with a fluorescent barcode. Hybridized DNA-CodeSet complexes were then aligned and immobilized in the nCounter Cartridges, which were transferred in the nCounter Digital Analyzer for data collection. We first assayed quality control checks on the raw code count data and then normalized them to a set of control probes representing invariant genomic regions. For each probe, the copy number was determined by calculating the ratio of normalized counts from a test sample to the counts of reference samples comprising a pool of 34 non-AD controls in this study. The ratio of counts was then rounded to the nearest integer to generate the final copy number calls. All the calculations were done using the R statistical analysis software (version 2.15.2) [20].

## Results

**Figure 1:**
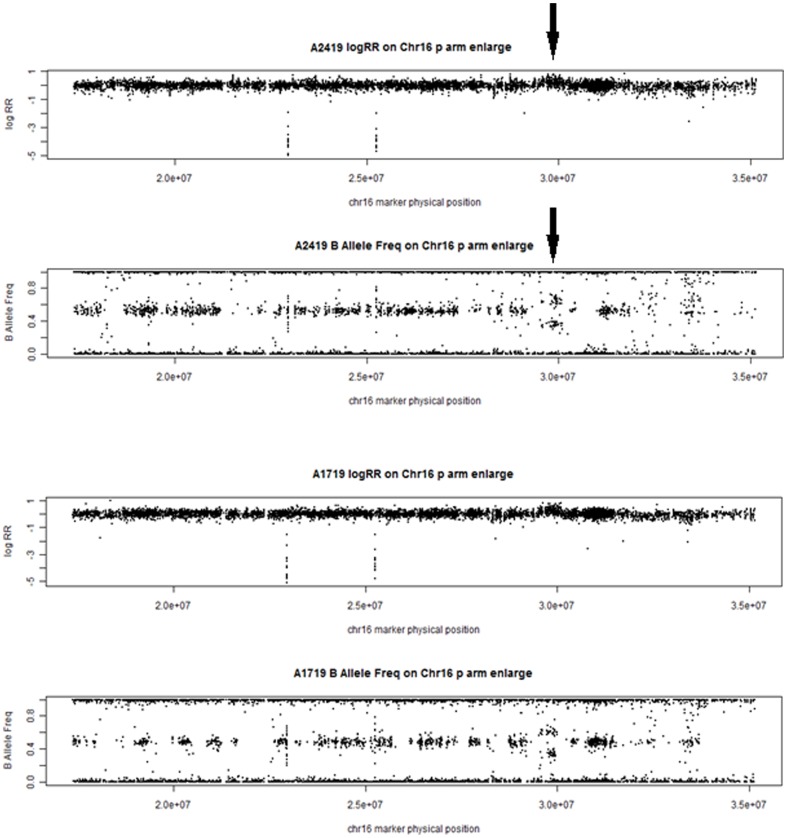
LRR and BAF plots for 16p11.2 duplication in two AD+P cases. LRR and BAF values for each probe are represented as dots. The black arrow delineates the position of the duplication (chr16: 29,557,553 to 30,107,434 bp). LRR values for the SNP and copy-number probes in the duplication increase and BAF values for the SNP probes cluster randomly around 0, 1/3, 2/3 and 1. In comparison, the flanking normal chromosomal regions have LRR values centered around zero with three BAF clusters (0, 1/2 and 1).

We detected a large (>0.5 Mb) and rare duplication CNV (copy number  = 3) on chromosome 16p11.2. It was present in 2 of 440 AD+P subjects but in none of 593 AD subjects with indeterminate psychosis, of 136 AD-P subjects, and of 855 non-AD controls (Table 2). The LRR and BAF plots of 16p11.2 duplication in two AD+P cases were shown in figure 1. The primary significance of this finding is that this large duplication of known pathogenicity is found at all in the individuals with AD+P. Statistical significance is highly dependent on the precise hypothesis being tested. Based on Fisher's exact test, the p-value for a difference between the frequency in AD+P subjects and AD-P subjects is 1.0. The p–value for the difference between AD+P and all other AD subjects is 0.14. The p-value for the difference between AD+P subjects and all other subjects (including non-AD) is 0.047. Each of these tests implies a different model for this duplication as a risk factor. It is not possible at the current sample size to say which of these models best fits the data. When type I error α is set at 0.05, our power to identify CNVs with frequency ≤0.5% in 440 AD+P subjects compared to 0% in all 1584 non-AD+P subjects is 65%. The power dropped to 18% to detect the same difference between 440 AD+P patients and all 729 other AD subjects, which may explain the borderline significant difference on 16p11.2 in the comparison of these two groups.

**Table 2:** Comparison of the duplication CNV on 16p11.2 identified in AD+P in this study to that reported in SCZ (McCarthy et al.'s, 2010).

	CNV Position	Number of total subjects	Number of subjects with CNV	Frequency of CNV
AD+P	29,554,843–30,105,652	440	2	0.0046
AD intermediate P		593	0	0
AD-P		136	0	0
No-AD controls		855	0	0
SCZ	29,557,498–30,107,355	4551	21	0.0046
No-SCZ controls		6391	2	0.0003

AD: Alzheimer's disease; AD+P: subjects with psychosis in Alzheimer's disease; AD-P: subjects with Alzheimer's disease without psychosis. SCZ: schizophrenia.

This duplication CNV almost completely overlaps with the previously reported 16p11.2 CNV region in autism and SCZ, spanning from 29,557,553 to 30,107,434 bp in SCZ and autism and from 29,554,843 to 30,105,652 bp in our study. The frequency of this duplication CNV in AD+P individuals (Table 2) is comparable to that reported in the combined samples from Shane's two large SCZ studies [22]. In the combined SCZ sample, 21 of 4551 cases (0.46%) carried this duplication compared with 2 of 440 AD+P cases in our study (0.46%).

The presence of the duplication was validated using the NanoString CNV Custom CodeSets (10 probe pairs). Table 3 lists the actual decimal copy number estimates generated by the ratio of counts from test samples to reference samples (34 non-AD individuals) and the final rounded integer copy number in two AD+P subjects with 16p11.2 duplication predicted by PennCNV as compared to random subjects without predicted CNVs. As expected, the final CNV calls for each of the 10 probes were 3 in duplication CNV carriers as compared to 2 in all other subjects, completely validating our findings.

**Table 3:** Actual CNV estimates and final CNV calls using NanoString CNV Custom CodeSets in two AD+P subjects with 16p11.2 duplication predicted by PennCNV as compared to random samples without predicted CNVs.

Subj ID	Genomic position (hg18)	Chr16 p11.2 Nanostring Probes									
	start	29564385	29568492	29610450	29727037	29775533	29868011	29885865	29940543	29995595	30050655
	end	29564462	29568583	29610524	29727118	29775619	29868017	29885959	29940612	29995679	30050742
1	AD+P **with** CNV	**3.4(3)** **	**3.1(3)**	**3.2(3)**	**3.0(3)**	**3.1(3)**	**3.0(3)**	**2.8(3)**	**3.3(3)**	**3.3(3)**	**3.0(3)**
2	AD+P **with** CNV	**3.2(3)**	**3.0(3)**	**2.9(3)**	**2.9(3)**	**2.7(3)**	**3.3(3)**	**3.3(3)**	**2.6(3)**	**3.1(3)**	**2.9(3)**
3	AD+P w/o* CNV	1.8(2)	1.7(2)	2.0(2)	2.0(2)	2.1(2)	1.7(2)	1.9(2)	1.6(2)	1.8(2)	1.9(2)
4	AD+P w/o CNV	1.9(2)	1.9(2)	2.0(2)	1.9(2)	1.8(2)	2.2(2)	2.0(2)	2.0(2)	1.8(2)	1.9(2)
5	AD+P w/o CNV	1.6(2)	1.8(2)	2.1(2)	1.9(2)	2.0(2)	2.1(2)	1.8(2)	2.3(2)	2.0(2)	2.0(2)
6	AD+P w/o CNV	2.2(2)	2.2(2)	2.4(2)	2.0(2)	2.1(2)	2.2(2)	2.2(2)	1.7(2)	1.9(2)	2.1(2)
7	AD+P w/o CNV	2.0(2)	2.2(2)	2.2(2)	2.1(2)	2.2(2)	1.9(2)	2.1(2)	2.1(2)	2.3(2)	2.2(2)
8	AD+P w/o CNV	1.9(2)	2.0(2)	2.0(2)	2.0(2)	1.8(2)	1.8(2)	1.8(2)	2.2(2)	2.0(2)	1.9(2)
9	AD intermediate P w/o CNV	1.9(2)	2.0(2)	2.1(2)	1.9(2)	1.9(2)	2.0(2)	1.8(2)	1.9(2)	2.0(2)	2.0(2)
10	AD intermediate P w/o CNV	2.2(2)	2.1(2)	1.9(2)	1.9(2)	2.2(2)	1.7(2)	2.0(2)	1.6(2)	2.1(2)	2.1(2)
11	AD intermediate P w/o CNV	1.8(2)	1.5(2)	1.6(2)	2.0(2)	1.7(2)	1.8(2)	1.7(2)	2.0(2)	1.9(2)	1.7(2)
12	AD-P w/o CNV	1.8(2)	1.8(2)	1.6(2)	2.0(2)	1.7(2)	1.8(2)	1.7(2)	2.0(2)	1.9(2)	1.7(2)
13	Non-AD w/o CNV	2.1(2)	2.0(2)	2.1(2)	2.1(2)	2.2(2)	1.7(2)	1.8(2)	1.8(2)	2.1(2)	2.2(2)
14	Non-AD w/o CNV	1.9(2)	1.9(2)	2.0(2)	2.0(2)	2.1(2)	2.1(2)	1.7(2)	2.2(2)	2.1(2)	1.9(2)

*w/o indicates subjects without predicted CNVs by PennCNV.

** Numbers in table represent actual CNV estimates whereas the numbers in parentheses indicate final CNV calls.

We did not find any CNVs greater than 50 Kb in the other 6 CNV regions that we investigated. We also did not find any deletions in the 16p11.2 region. Deletions in this region have been associated with mild mental retardation, autism, and other non-behavioral phenotypes [23]–[25].

## Discussion

To our knowledge, we are the first to report that patients with AD+P share a rare risk CNV on chromosome 16p11.2 with SCZ and autism patients. The frequency of the duplication in our AD+P cohort is 0.46% (2 out of 440 AD+P), which is similar to that previously reported in SCZ. This frequency estimate is very approximate due to the small sample size; however, a larger replication cohort is warranted for further investigation.

Several discrete phenotypes, such as developmental delay, intellectual disability, behavioral problems, autism, schizophrenia, and bipolar disorder have been previously shown to be associated with the microduplication of 16p11.2 and are considered as 16p11.2 duplication syndromes [22], [24]–[27]. Further, a newly published paper [28] revealed a common variant (rs4583255) located at 16p11.2 that increased the risk of both SCZ and bipolar disorder with similar odds ratios. Since the 16p11.2 duplication has been previously reported in the above psychiatric disorders, we examined whether comorbidity existed in these two AD+P patients. Medical history and examination showed these two AD+P patients had no personal history of, nor any identified family history of, SCZ, bipolar disorder and autism.

Our finding potentially extends the range of 16p11.2 duplication syndromes to psychosis in Alzheimer's disease. Pleiotropy of CNVs has been established [29], however, the genetic etiology of the pleiotropic effects is unknown. It has been proposed by DeMichele-Sweet and Sweet [30] that neurodevelopmental disorders and neurodegenerative disorders (e.g., AD) may share some common disease modifier genes, which may be involved in the development of psychosis in different disorder processes. Our findings partially support one of their hypothesized pathways. It is possible that distinct psychiatric disorders share the same or similar genetic factors affecting the same or neighboring (and maybe functionally-related and/or interacting) genes, perhaps influenced by different genetic and/or environmental modifiers. The quite different disease onset time between patients with AD+P and some patients with neurodevelopmental disorders, such as autism, may be partially related with incomplete but substantial (∼30–50%) penetrance of 16p11.2 duplication [22] or genetic mosaicism of the 16p11.2 region [31].

Microduplication of 16p11.2 lies in a chromosomal rearrangement hotspot [31] and dosage-sensitive loci [32], which contain about 25 genes (*SPN, QPRT, JCLN, KIF22, MAZ, PRRT2, MVP, CDIPT, PIS, SEZ6L2, ASPHD1, KCTD13, FKSG86, TMEM219, TAOK2, HIRIP3, INO80E, DOC2A, ALDOA, PPP4C, TBX6, YPEL3, GDPD3, MAPK3, and CORO1A*). Most of these 25 genes are expressed in brain, among them several genes have been found to be involved in Alzheimer's disease, including *SPN* (sialophorin, also named as *CD43*), *CORO1A* (coronin, actin binding protein, 1A), *QPRT* (quinolinate phosphoribosyltransferase), *MAZ* (MYC-associated zinc finger protein), and *MAPK3* (mitogen-activated protein kinase 3, also named as *ERK1*).

Expression change of *SPN* in human microglia was reported in Alzheimer's disease [33]; Specific carbonyl level of *CORO1A* was reduced during the treatment to reduce Amyloid β-peptide levels in mouse model [34]. *QPRT* is the enzyme responsible for quinolinic acid (QUIN) turnover in the kynurenine pathway of tryptophan degradation. Increased QUIN has been reported in AD brain [35]. Increased concentrations of kynurenine pathway metabolites have also been reported for disorders involving psychosis, including schizophrenia and bipolar disorder [36]. *ZF87*/*MAZ* and *FAC1* co-localize to pathologic structures in Alzheimer's disease brain. The interaction of these two proteins was involved in gene regulation in neurodegeneration [37], [38]. *MAZ* was also identified as a blood biomarker in schizophrenia [39]. Accumulated evidence have shown that up-regulation of *MAPK3* (*ERK1*) are associated with the progression of Alzheimer's disease [40]–[44]. In addition, expression of *MAPK3* is significantly associated with a locus (rs4583255[T]) on 16p11.2 which shows genome-wide significant association with psychosis, including schizophrenia, bipolar disorder and related psychoses by a recent GWAS (genome-wide association study) in a large cohort [45]. It also modifies the effect of *KCTD13* on zebrafish head size [32].

*KCTD13* (potassium channel tetramerization domain containing 13) was revealed recently by Golzio et al [32] as a major driver for the neurodevelopmental phenotypes associated with the 16p11.2 CNV, and 16p11.2 duplication was associated with microcephaly phenotype in zebrafish embryos with MAPK3 and MVP named as possible modifiers. They further reported that microcephaly was caused by decreased proliferation of neuronal progenitors with concomitant increase in apoptosis in the developing brain by zebrafish and mouse embryos. This may explain a common effect for these three distinct phenotypes. However, we cannot exclude the possibility that the genes contributing to psychosis in AD are different from the genetic effects leading to any of the other disorders. It is also unknown if this CNV plays a heterogenetic role in the development of AD and subsequent psychosis or it modifies risk of psychotic symptoms in the presence of AD pathology. Given the low frequencies of the duplication, it may not be possible to discern these two possibilities.

Although we validated this 16p11.2 duplication using the NanoString technology, future studies are warranted to replicate our findings in large independent samples. Also, since this duplication is about 550kb long and contains about 25 genes, and its effect on functionality of those 25 genes is unclear, more work is necessary to further refine this important region and to identify the disease causing gene(s). The Simons VIP Consortium [46] has recently launched a project to study the genetic causes of neuropsychiatric disorders with primary focus on the 16p11.2.

It should be noted that even though the 16p11.2 duplication may play an important role in development of psychosis of Alzheimer's disease, as in autism and schizophrenia, its frequency is low (0.46%) compared to the disease frequency (40∼60% of AD patients who develop psychosis), which suggests that 16p11.2 duplication can be responsible only for a small fraction of psychiatric morbidity. A potential limitation of this study is that it has restricted power to detect very rare CNVs in AD+P. We consider our study primarily as a case report; the estimation of frequency, p-value and/or power will require larger cohorts.

Besides those 7 CNV regions previously reported to be shared across autism and SCZ, a newly published paper [47] identified a duplication overlapping the p21 Protein-Activated Kinase (*PAK7*) as a risk factor for psychosis. We did not find duplications/deletions overlapping this gene in our study.

In conclusion, although rare, the 16p11.2 duplication we have identified in AD+P may help to understand an underlying mechanism for the development of psychosis in AD. In addition, this CNV has the potential to be used in clinical practice for screening, diagnosis, and classification of psychosis in AD.
